# Supplementary material for: Predicting visual function by interpreting a neuronal wiring diagram
Source: Nature. 2024 Oct 2;634(8032):113–23. doi: 10.1038/s41586-024-07953-5 (PMC11446822; doi:10.1038/s41586-024-07953-5)
Supplement: Supplementary file 6 — CRF and ERF predictions for individual TmY4 and TmY9 cells. Analogous to Supplementary Data 3, but for TmY target types. Shown are the top four monosynaptic pathways, the strongest pathway passing through each of the top ten intermediary types (ranking from Extended Data Fig. 7), and the trisynaptic pathway Tm1–TmY–Dm3–TmY (see the section entitled Prediction of spatial normalization). [file 41586_2024_7953_MOESM6_ESM.zip › DataS4/TmY9qΓèÑ/720575940634633753.pdf]

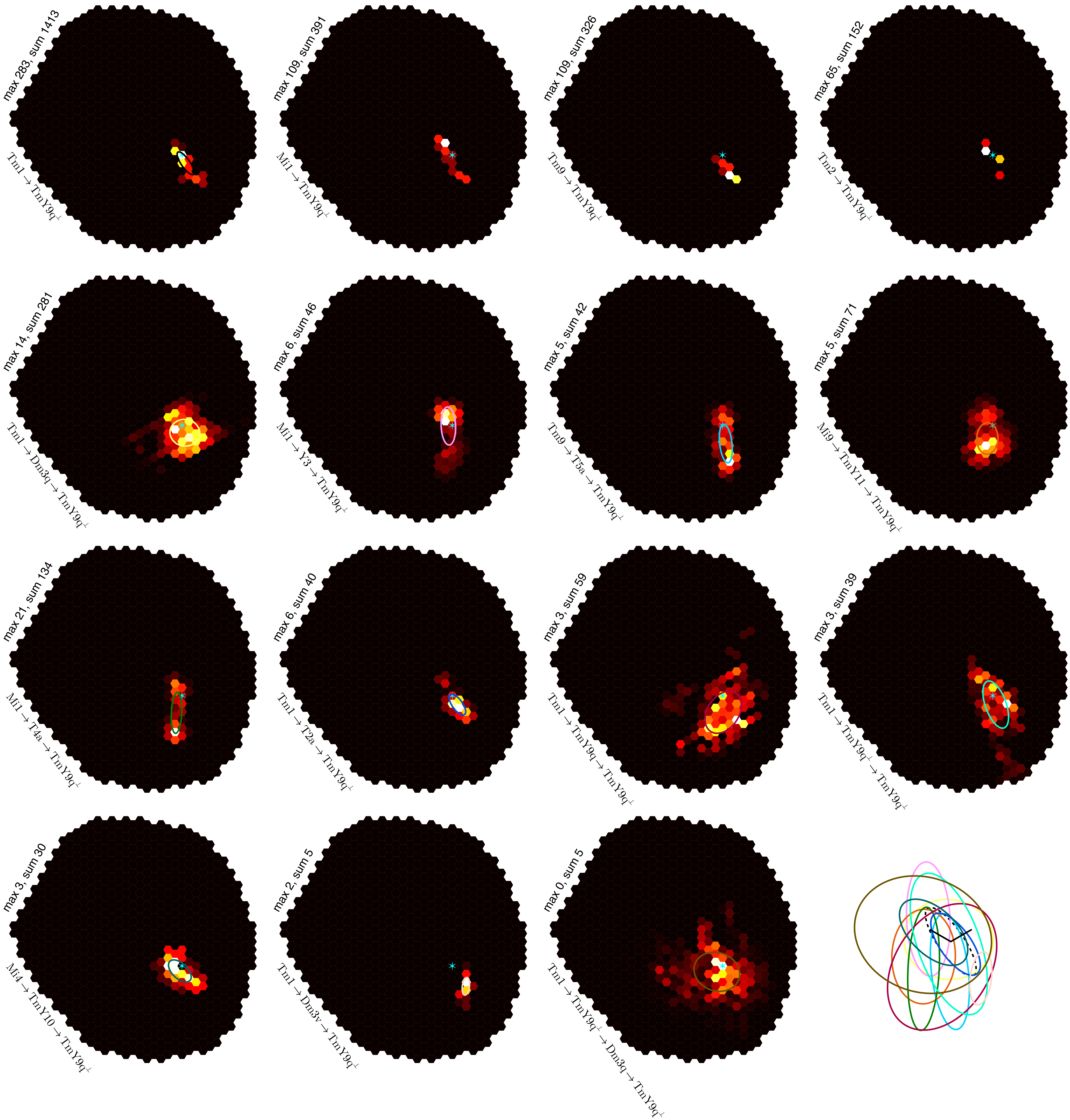
max 283, sum 1413
Tm1 → TmY9q⊥
max 109, sum 391
Mi1 → TmY9q⊥
max 109, sum 326
Tm9 → TmY9q⊥
max 65, sum 152
Tm2 → TmY9q⊥
max 14, sum 281
Tm1 → Dm3q → TmY9q⊥
max 6, sum 46
Mi1 → Y3 → TmY9q⊥
max 5, sum 42
Tm9 → T5a → TmY9q⊥
max 5, sum 71
Mi9 → TmY11 → TmY9q⊥
max 21, sum 134
Mi1 → T4a → TmY9q⊥
max 6, sum 40
Tm1 → T2a → TmY9q⊥
max 3, sum 59
Tm1 → TmY9q⊥ → TmY9q⊥
max 3, sum 39
Tm1 → TmY9q⊥ → TmY9q⊥
max 3, sum 30
Mi4 → TmY10 → TmY9q⊥
max 2, sum 5
Tm1 → Dm3v → TmY9q⊥
max 0, sum 5
Tm1 → TmY9q⊥ → Dm3q → TmY9q⊥
